# Supplementary material for: Gene Expression of Transient Receptor Potential Channels in Peripheral Blood Mononuclear Cells of Inflammatory Bowel Disease Patients
Source: J Clin Med. 2020 Aug 14;9(8):2643. doi: 10.3390/jcm9082643 (PMC7547374; doi:10.3390/jcm9082643)
Supplement: Supplementary file 1 [file jcm-09-02643-s001.pdf]

Supplementary Materials

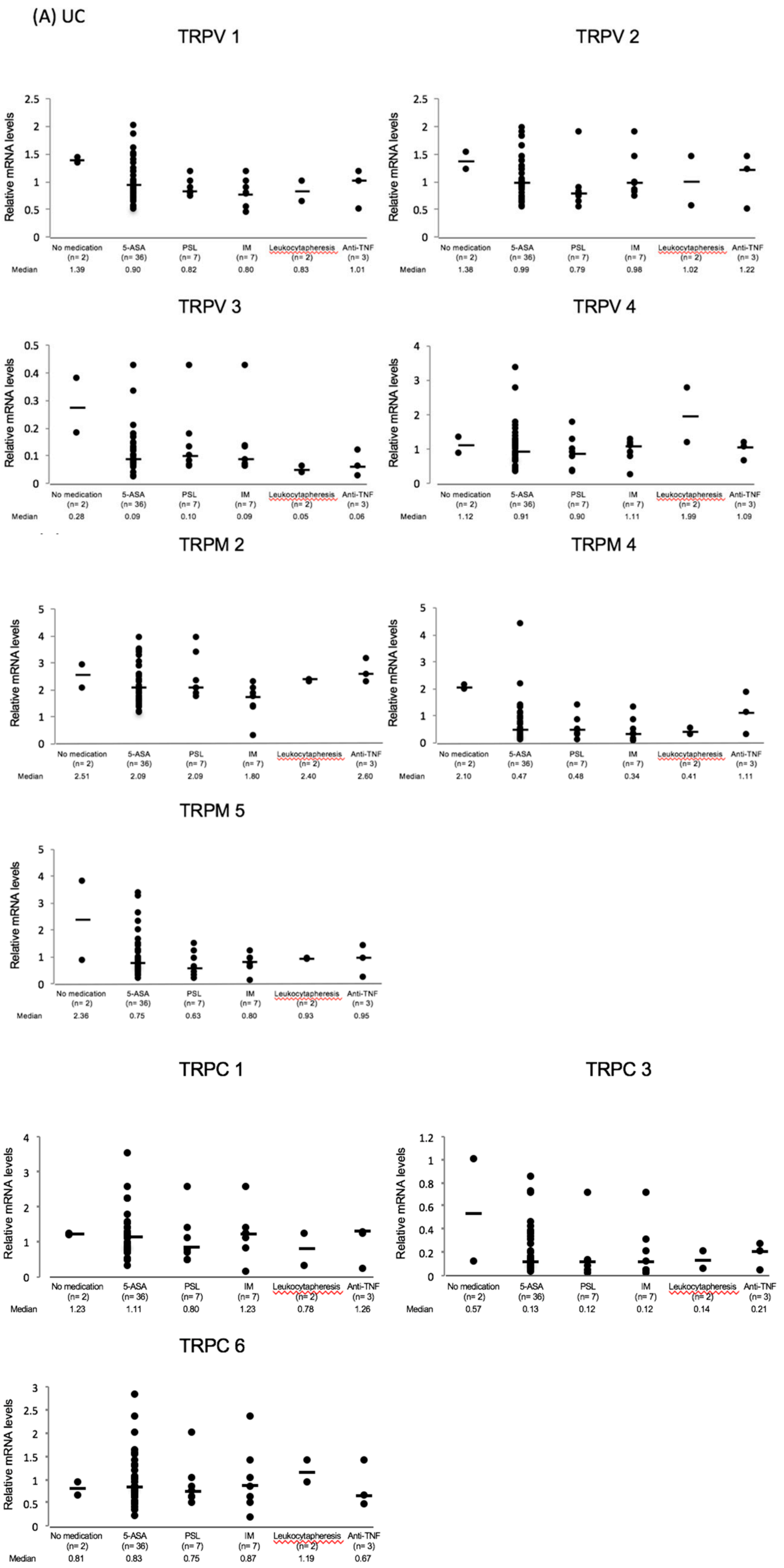

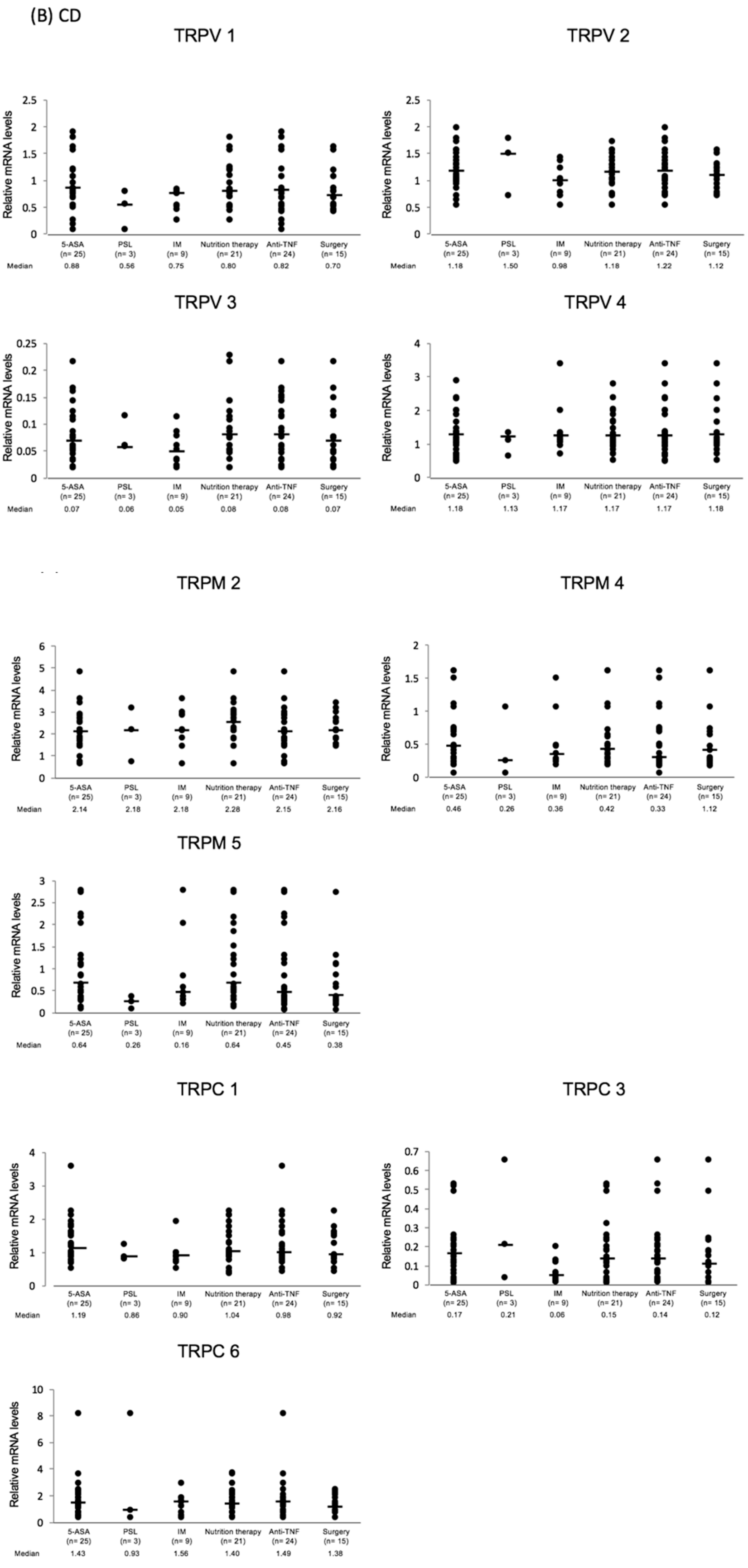

Figure S1: Comparison of TRP channel expression according to the current use of medications in patients with (a) ulcerative colitis (UC) and (b) Crohn's disease (CD) (b). Some of the patients received more than one category

of treatment. The bar represents the median. 5ASA: 5-aminosalicylic acid; PSL: Prednisolone; IM: Immunomodulator; LCAP: Leukocytapheresis; TNF: Tumor necrosis factor.
